# Antagonism of Therapeutic Agents

**Published:** 1878-05-20

**Authors:** 


### ANTAGONISM OF THERAPEUTIC AGENTS.

---

Dr. J. Milner Fothergill, in the Philadelphia *Medical Times*, contributes a very interesting letter, from which the following extract is made:

"At the risk of being charged with egotism and some vanity, I may refer to the subject-matter of an essay to which has just been awarded the Fothergillian gold medal of the Medical Society of London. The subject of the essay was "The Antagonism of Therapeutic Agents," a matter of great interest at the present time, and one which will wax in importanse in the future. With the exception of a confused impression as to the antagonism of opium and belladonna, our knowledge has been almost confined to chemical antidotes and their utility in poisoning—the use of sulphuric acid and of iodide of potassium in chronic lead-poisoning being an example. But recent researches have demonstrated that many agents have a physiological antagonism, which may be utilized practically. Thus, Prof. Frazer, of Edinburgh, worked out the antagonism of calabar bean and belladonna in the most thorough and efficient manner. He showed that not only could minimum lethal doses be successfully antagonized, but that considerably larger doses could be met successfully by correspondingly larger doses of the physiological antagonist. He made most exact observations as to the effect of calabar bean upon the respiration and the circulation, and showed how, when both were failing conspicuously, the administration of atropia restored them completely. In fact, the interest of the experiments performed so far has lain around those rhythmically discharging centres which preside over the respiration and the circulation. The centres for the respiration are situated in the medulla oblongata, the nœud vital of Flourens; while those of the circulation are essentially the ganglia which lie in the septa of the auricles and betwixt the auricles and ventricles. In both these motor centres there is an accumulation of energy which explodes rhythmically and sets the muscular mechanism in action. It is these centres upon whose activity life depends, and it is the effects of toxic agents upon these centres which make them dangerous to life. The unconsciousness produced by opium is in itself of little importance: it is the failure of the respiration first, and then of the heart next, which constitutes the real danger; and paralysis of the nerve-centres of these systems is the action of opium in toxic doses which is to be feared. Oscar Liebreich first observed that strychnia and chloral possessed a powerful antagonistic action, which might be utilized in practice; and there is a well-known case, published by Dr. Levinstein, where an overdose of chloral had been taken, and which recovered, after most grave symptoms had manifested themselves, by the use of nitrate of strychnia injected subcutaneously. Many experiments were performed by the Edinburgh committee of the British Medical Association, presided over by the late Prof. J. Hughes Bennett, by which the antagonism of various agents was demonstrated and proved.

Then Dr. Crichton Browne demonstrated how the convulsions produced by picrotoxine, the active principle of the coculus Indicus, could be controlled by chloral. It was very interesting to watch two rabbits to each of which a lethal dose of picrotoxine had been administered, but to one the antagonistic dose of chloral had also been given. The first was subject to recurrent attacks of fearful spasm, very much like strychnia spasms, including opisthotonos, culminating in a terrible final convulsion; while the other lay peacefully before the fire, wrapt in chloral sleep, an occasional slight twitch alone indicating the presence of the picrotoxine. But if this second rabbit were wakened out of its chloral sleep, then a picrotoxine fit would come on, resembling those in the rabbit without chloral; before a second convulsion could come on, the chloral narcosis had resumed its sway, and the animal slept on undisturbed, to awaken up well alongside the stiff corpse of its less fortunate companion. It was evident that discharges from large motor areas were excited by the action of the picrotoxine, and that chloral could restrain them if given in sufficient quantity. These experiments have had much to do in deciding the present large resort to chloral in asylums to control maniacs and general paralytics in their recurring infuriated outbreaks of violence.

Then a series of experiments were performed by the writer to test the antagonism of aconite and digitalis in warm-blooded animals. It was soon apparent that in the rabbit and guinea-pig digitalis did not sufficiently antagonize the effect of the aconite upon the respiration to be useful. It exercised some effect if given from five to nine hours before the administration of aconite. It, however, maintained the action of the heart, which was found contracted firmly after death; but it did not prevent efficiently the action of the aconite upon the respiration. The belladonna was tried and found to be a perfect antidote, as might have been expected from its well-known action as a stimulant to the respiratory centres and to the cardiac ganglia. The animals, expiring with respiratory gasps at gradually lengthening intervals, began to respire more forcibly after the administration of belladonna, and more quickly, until normal respiration was regained. The atropia was effective in saving life up to sixteen minutes after the injection of the aconite—a long time in aconite-poisoning, but if delayed longer it prolonged life but could not save it. Then strychnine was tried, and was found most effective, the animals recovering swiftly, only to die in the expiratory spasm of strychnia when perfectly recovered from the aconite-paralysis. At this interesting point the Anti-Vivisection Act came into force, and brought to a close a series of experiments which are well worth carrying out by some investigator in a land less hampered than is Great Britain in the matter of scientific inquiry involving experimentation upon animals.

These observations as to the effects of certain toxic agents upon the action of other toxic agents tell us much that can be made practically useful. In the first place, this physiological antagonism has been utilized in cases of poisoning, and strychnia-poisoning has been several times successfully treated by chloral, as well as the opposite in Dr. Levinstein's case. Dr. Dobie, of Keighley, used digitalis successfully in a case of aconite-poisoning, where the man was very far gone. Here the digitalis must have exercised some influence upon the respiration as well as the circulation, or else an effect upon the circulation is soon felt by the respiration, so closely are these two centres linked together. In a recent case the writer gave a grain of sulphate of atropia at once, subcutaneously, to a woman far advanced in opium-poisoning, with the effect of an early restoration of the respiration, which was notably failing while the pulse kept steady. Without previous acquaintance with the effect of the administration of a counter-poison to animals dying from the toxic effects of another poison previously administered, probably some hesitation might have been experienced as to the large dose adopted. The result, however, justified the size of the dose completely. In fact, our acquaintance with the subject of the antagonistic action of certain poisons must exercise a potent influence over the future of toxicology. Probably it will be found that the best plan of treating opium-poisoning will be to empty the stomach thoroughly, and then to inject subcutaneously a fourth or third of a grain of atropia before the respiration has begun to fall; after that, to put the patient to bed, and watch assiduously the respiration, the circulation, and the body-temperature. If the respiration should still show indications of failing, to inject a second dose of atropia of equal size would be the best thing to be done, or even more if required. This would be much more effective than dragging the patient about and administering strong coffee, and would enable the medical attendant to take minute observations whose value we may not yet be in a position to estimate. A further outcome of such a plan of treating opium-poisoning would be that we would soon learn how far atropia could be trusted to antagonize the action of opium upon the centres of respiration and circulation, and how little it affects the action on the sensorium. There exist excellent grounds for believing that by such combination we will be enabled to give, without anxiety as to the result, much larger doses of opium or morphia than have hitherto been thought safe, in cases of severe pain, or in the fearful cough of some cases of softening tubercle. In all cases the toxic action of the opium upon the respiration should be made the ground for action, and not any change in the pupil, as has hitherto been done. The pupil is a dubious and unsafe guide, for it may be dilated by atropine even when the dose is utterly ineffective to arrest the opium-poisoning, as was seen in a case lately recorded by Dr. Paget, of Cambridge.

Other uses of such advancing acquaintance with the effects of toxic agents upon the respiration are developing themselves. When the respiration is embarrassed in asthma we know that belladonna often gives great relief, as it also does in whooping-cough. But in order to give a remedy with some approach to rational certitude in either of these maladies, it is well to note the general condition of the respiration and be guided in the selection of a remedy accordingly. If it be found excited give an agent which calms the nœud vital; if depressed a stimulant agent like belladonna is indicated. In a little time we shall prescribe with considerably more accuracy in these neuroses of the respiration. Then, too, in chronic bronchitis with emphysema, strychnia and belladonna are very useful, and in the bulk of cases give great relief. In more acute conditions they give much promise, and a friend of mine recently pulled through successfully, by the use of strychnia, a case of capillary bronchitis which seemed as if it must necessarily end fatally. It was with much satisfaction the writer read the paper by Dr. Reinhard Weber, in the Philadelphia *Medical Times* for February 2d, on the use of belladonna in collapse, and the use of agents acting powerfully upon the respiration and circulation must obtain extensively in the future in temporary asthenic conditions where life is gravely threatened without death being unavoidable, and where a slight matter even may settle the question of life and death. But the subject cannot be pursued further within the limits of a letter.

